# The NAC transcription factor *ClNAC68* positively regulates sugar content and seed development in watermelon by repressing *ClINV* and *ClGH3.6*

**DOI:** 10.1038/s41438-021-00649-1

**Published:** 2021-10-01

**Authors:** Jinfang Wang, Yanping Wang, Jie Zhang, Yi Ren, Maoying Li, Shaowei Tian, Yongtao Yu, Yi Zuo, Guoyi Gong, Haiying Zhang, Shaogui Guo, Yong Xu

**Affiliations:** National Watermelon and Melon Improvement Center, Beijing Academy of Agricultural and Forestry Sciences, Key Laboratory of Biology and Genetic Improvement of Horticultural Crops (North China), Beijing Key Laboratory of Vegetable Germplasm Improvement, Beijing, 100097 China

**Keywords:** Auxin, Fruiting, Seed development

## Abstract

NAC (NAM, ATAF1/2, and CUC2) transcription factors play important roles in fruit ripening and quality. The watermelon genome encodes 80 NAC genes, and 21 of these NAC genes are highly expressed in both the flesh and vascular tissues. Among these genes, *ClNAC68* expression was significantly higher in flesh than in rind. However, the intrinsic regulatory mechanism of *ClNAC68* in fruit ripening and quality is still unknown. In this study, we found that ClNAC68 is a transcriptional repressor and that the repression domain is located in the C-terminus. Knockout of *ClNAC68* by the CRISPR-Cas9 system decreased the soluble solid content and sucrose accumulation in mutant flesh. Development was delayed, germination was inhibited, and the IAA content was significantly decreased in mutant seeds. Transcriptome analysis showed that the invertase gene *ClINV* was the only gene involved in sucrose metabolism that was upregulated in mutant flesh, and expression of the indole-3-acetic acid-amido synthetase gene *ClGH3.6* in the IAA signaling pathway was also induced in mutant seeds. EMSA and dual-luciferase assays showed that *ClNAC68* directly bound to the promoters of *ClINV* and *ClGH3.6* to repress their expression. These results indicated that *ClNAC68* positively regulated sugar and IAA accumulation by repressing *ClINV* and *ClGH3.6*. Our findings provide new insights into the regulatory mechanisms by which NAC transcription factors affect fruit quality and seed development.

## Introduction

Fruit ripening is a complex process, and multiple metabolic changes, including changes that affect sugar, organic acids, and pigments, occur during fruit ripening and postharvest storage^1^. The network of underlying molecular mechanisms that directly regulate fruit ripening and determine fruit quality has been revealed; this network includes hormones and signaling pathways, transcriptional regulators, and other regulatory elements^2^. Watermelon (*Citrullus lanatus*) is one of the most popular fruits^3^, and it is important to explore the intrinsic mechanism regulating metabolic changes to improve fruit quality.

Numerous studies have shown that enzymes involved in sugar metabolism, including sucrose synthase (Sus), sucrose phosphate synthase (SPS), and invertases (INVs), play important roles in regulating sucrose accumulation in fruit^4^. Among these enzymes, Sus or INVs catalyze the hydrolysis of sucrose into fructose and glucose, and the activity of these two enzymes shows a negative correlation with sugar content^5^. Recently, studies revealed that transcription factors mediate the regulation of these important enzymes to determine the sugar content in seeds and fruit. In maize, the endosperm-specific transcription factor Opaque2 (O2) was found to activate the expression of *sus1* and *sus2* and result in a higher soluble sugar content in the *o2* mutant^6^. In pitaya, HpWRKY3 was found to activate the expression of *HpINV2* and *HpSuSy1* to regulate sugar accumulation, but there was no transgenic evidence to further determine this relationship^7^. In our previous study, we found that the total sugar and sucrose contents increased, while the expression of Sus and acid INV showed a significant negative correlation with sucrose content during watermelon fruit ripening^8^. However, the regulators of Sus and acid INV during watermelon fruit and seed development have not been well illustrated.

Plant hormones and hormone signal transduction pathways play important roles in regulating seed germination, plant growth, development, and fruit ripening^9^. In cucumber, the IAA content was found to show a positive correlation with fruit size^10^. In peach, suppression of *PpYUC11* was found to decrease the IAA level and result in increased fruit fitness^11^. Another important IAA-amino synthetase (GH3) gene in the IAA signaling pathway catalyzes the binding of amino acids to IAA and results in inactive IAA and a reduction in the free IAA content^12^. In apple, overexpression of *MsGH3.5* was found to result in dwarf plants and fewer adventitious roots, and the IAA and cytokinin contents were significantly reduced^13^. In kiwifruit, silencing *AcGH3.1* by VIGS increased fruit firmness during kiwifruit postharvest storage. Moreover, the IAA content was increased during seed development, and a higher IAA content affected seed size^14^. In soybean, the IAA content was found to be significantly higher in variants with larger seeds than in variants with smaller seeds^14^. In rice, overexpression of *OsYUC2* was found to increase grain yield by increasing the IAA level^15^. However, how the IAA and IAA signaling pathways regulate fruit and seed development in watermelon is still unknown.

NACs (NAM, ATAF1/2, and CUC2) constitute one of the largest plant-specific transcription factor families. NACs are involved in regulating processes of plant development, including root elongation, leaf senescence, and fruit ripening^16,17^. Overexpression of *MdNAC42* in apple calli was found to activate the expression of flavonoid pathway genes and induce anthocyanin accumulation^18^. In tomato, overexpression of *SlNAP2* was found to accelerate leaf senescence by increasing ABA biosynthesis genes and the expression of chlorophyll degradation genes^19^. Furthermore, although the sugar content was found to be significantly reduced in OE lines and induced in knockout lines, the mechanism of *SlNAP2* in the regulation of sugar metabolism is still unknown^19^. Most previous studies focused on NAC-mediated regulation of seed germination via the ABA pathway, while a few studies suggested that NAC regulates seed germination through the IAA pathway. In rice, overexpression of *OsNAC2* was found to inhibit root elongation by affecting the expression of auxin- and cytokinin-responsive genes^20^. In Arabidopsis, the NAC transcription factor NTM2 was found to negatively regulate seed germination under salt stress by upregulating *IAA30* expression^21^. In watermelon, 80 *NAC* transcription factors and 21 *ClNACs* were found to be expressed in both vascular tissues and fruit, and these 21 *ClNACs* might regulate watermelon fruit ripening or quality^22^. Among these NAC genes, *ClNAC68* (Cla97C03G059250, Cla019693 in version 1 of the watermelon genome) was found to be highly expressed in the later stages, and its expression was significantly higher in flesh than in rind. These results indicated that *ClNAC68* might play an important role in regulating watermelon fruit ripening. However, the intrinsic mechanism of ClNACs in the regulation of fruit ripening, fruit quality, and seed development is still unknown.

In this study, we characterized the NAC gene *ClNAC68* and explored its function in fruit and seeds. Knockout of *ClNAC68* by the CRISPR-Cas9 system decreased the fruit sugar content and delayed seed maturation. The seed germination rate and root elongation were also decreased in mutant lines, and exogenous IAA treatment partially reversed the inhibition. The biological function and the regulatory network of *ClNAC68* were comprehensively analyzed, and the findings revealed that *ClNAC68* plays a critical role in sugar accumulation and seed development.

## Materials and Methods

### Plant transformation and plant growth conditions

To investigate the function of *ClNAC68*, the CRISPR/Cas9 vector pBSE401 was used to generate gene knockout plants for further analysis. Two target sites were designed on the website (https://www.addgene.org/crispr/) and incorporated into forward and reverse PCR primers (DT1 and DT2, respectively) as described by Tian et al.^23^ (Supplemental Table S1). Then, the pBSE401-*ClNAC68* construct was transferred into *Agrobacterium tumefaciens* strain EHA105 and used for transformation. Plant transformation was performed according to the method described by Tian et al.^23^ PCR was used to identify the homozygous mutant and Cas9-free generations. The primers used are listed in Table S1. All seedlings of mutant lines and WT lines were grown in a plastic greenhouse under natural growing conditions. The phenotypes of the mutant and WT lines were observed at 18 and 26 days after pollination (DAP).

### Subcellular localization

The full coding sequence (CDS) of the gene without the stop codon was inserted into the pYBA1332 vector, which contained a GFP tag. The expression construct and empty vector were transferred into watermelon leaf protoplasts. The methods used for protoplast extraction and transformation were described in our previous study^24^. After 24 h of transformation, the protoplasts were incubated with DAPI solution (Sigma, USA; 1 mg/mL) in the dark for 5 min and observed by confocal microscopy (Zeiss, LSM700 laser-scanning confocal microscope, Germany).

### Transcriptional assay

For the yeast two-hybrid assay, the full-length NAC domain fragment and transcription domain fragment of *ClNAC68* were cloned into the pGBKT7 vector (Table S1). All constructs were transformed into the yeast strain Y2H-Gold and cultured in SD-T and SD-TH selective media according to the manufacturer’s instructions (Clontech, USA) at 28 °C for three to five days. pGBKT7-P53 and pGBKT7 were used as the positive and negative controls, respectively. The experiment was performed in triplicate.

For measurement of transient luciferase activity, different fragments of *ClNAC68* were inserted into the GAL4DB vector. The empty GAL4DB vector and herpes simplex virus 16 (VP16) were used as the negative and positive controls, respectively. Then, these constructs functioned as effectors and were transiently transformed into watermelon leaf protoplasts with a luciferase reporter vector. Renilla luciferase was used as the internal control. The plasmid ratio used was pTRL:reporter:ClNAC68/none/VP16) = 1:6:6. Furthermore, these constructs were coexpressed with VP16 in watermelon leaf protoplasts with the reporter and internal control, and the plasmid ratio used was pTRL:reporter:ClNAC68/actin/none:VP16 = 1:6:6:6, following the method described in a previous study by Wei et al.^25^^.^ The watermelon actin gene *Cla97C02G026960* was used as the noninteractive control. The dual-luciferase activity was measured according to the manufacturer’s instructions (Vazyme Biotech, China, Nanjing). The experiment was performed with six biological replicates.

### Measurement of sugar content and invertase activity

The brix of WT and *clnac68* mutant fruits was measured with a hand-held ATC-1E refractometer (ATAGO, Tokyo). Sugar content was measured by Majorbio (http://www.majorbio.com/) based on the Thermo ICS5000 platform. Invertase activity was measured as described in our previous study^8^. Three biological replicates were performed.

### Measurement of phytohormone content

Free IAA and ABA were measured with an ESI-HPLC-MS system. IAA and ABA standards (Sigma, USA) were used to determine the retention time and mass spectra. Free IAA and ABA were measured following the method described in our previous study^26^ in a biological mass spectrometry laboratory at China Agricultural University. Three biological replicates were performed.

### Seed germination and root elongation assays

For the seed germination assay, WT and *clnac68* mutant seeds were germinated under normal and exogenous IAA and ABA treatment. The seeds were soaked for 4 h in 50 °C water and were then transferred into culture dishes with filter paper containing 5 mL of distilled water, 10 μM IAA (Sigma, USA), and 10 μM ABA (Sigma, USA). All seeds were induced to germinate at 28 °C in the dark and were observed at 24, 36, 48, 60, 72, 84, and 96 h to calculate the seed germination rate (germinated seeds/total seeds). Sixty seeds were used for each treatment condition, and three biological replicates were performed. For the root elongation assay, after 48 h of germination, germinated seeds were selected to measure the root length. All seeds were grown vertically on filter paper containing 5 mL of distilled water or 10 μM IAA (Sigma, USA). After 5 days, the root lengths of WT and *clnac68* mutant seeds were quantified.

### Transcriptome analysis and qRT-PCR analysis

Intact leaves in the middle of watermelon vines and healthy roots were collected from 10 to 34 days after the pollination stage. The center flesh and seeds were collected and immediately frozen in liquid nitrogen for further use. Three seedlings or fruits were collected for each analysis.

For transcriptome analysis, 12 RNA libraries were built with a TruSeq^TM^ RNA Sample Prep Kit (Illumina, USA) and sequenced on the Illumina HiSeq 4000 platform using the paired-end 150 bp read mode. Gene expression levels were normalized to transcripts per million reads (TPM) values. The differentially expressed genes (DEGs) (*p*-adjust < 0.05 and | log2FC | > = 1) between the *clnac68* mutant lines and WT lines were selected for further research. The data were analyzed on the free online platform of the Majorbio I-Sanger Cloud Platform (www.i-sanger.com).

For qRT-PCR analysis, cDNA was reverse transcribed with FastKing gDNA Dispelling RT SuperMix (Tiangen Biotech, Beijing, China). qRT-PCR was performed in a LightCycler 480 RT-PCR system (Roche, Switzerland) with specific primers (Supplemental Table S1). The watermelon actin gene *Cla97C02G026960* was used as the internal control.

### Electrophoretic mobility shift assay (EMSA)

The full-length *ClNAC68* sequence without a stop codon was fused to an MBP tag and inserted into the vector pMCSG7-MBP-His, and the ClNAC68 protein was purified by His-agarose affinity chromatography. The biotin-labeled probes were the promoters of *ClINV or ClGH3.6* containing NAC binding sites, and in the mutant probes, the NAC core binding sites ‘CGTG(A)’ were replaced with continuous ‘AAAA’ sequences. The nonbiotin-labeled segments of the same sequences were used as competitors (Supplemental Table S1). EMSA was performed according to the protocol of the Light Shift Chemiluminescent EMSA Kit (Thermo Fisher Scientific, Shanghai, China). The MBP protein was used as the negative control.

### Measurement of transient dual-luciferase activity

The full-length *ClNAC68* sequence was inserted into the pGreenII 62-SK vector as an effector, and the promoters of *ClINV* and *ClGH3.6* containing NAC binding sites were inserted into pGreenII 0800-LUC as the reporter (Supplemental Table S1). All these constructs were transformed into *Agrobacterium tumefaciens* strain GV3101, and the effector was transfected with different reporters into tobacco leaves. The pGreenII 62-SK empty vector was used as the negative control. Luciferase activity was measured following the protocol of the Dual-Luciferase Reporter Assay Kit (Vazyme Biotech, Nanjing, China). Five biological replicates were performed.

### Statistical analysis

The data were analyzed by Student’s *t* test, and the significance of differences relative to WT plants or negative controls was evaluated using GraphPad 8.0.

## Results

### *ClNAC68* was highly expressed in flesh and proved to be a transcriptional repressor

**Fig. 1:**
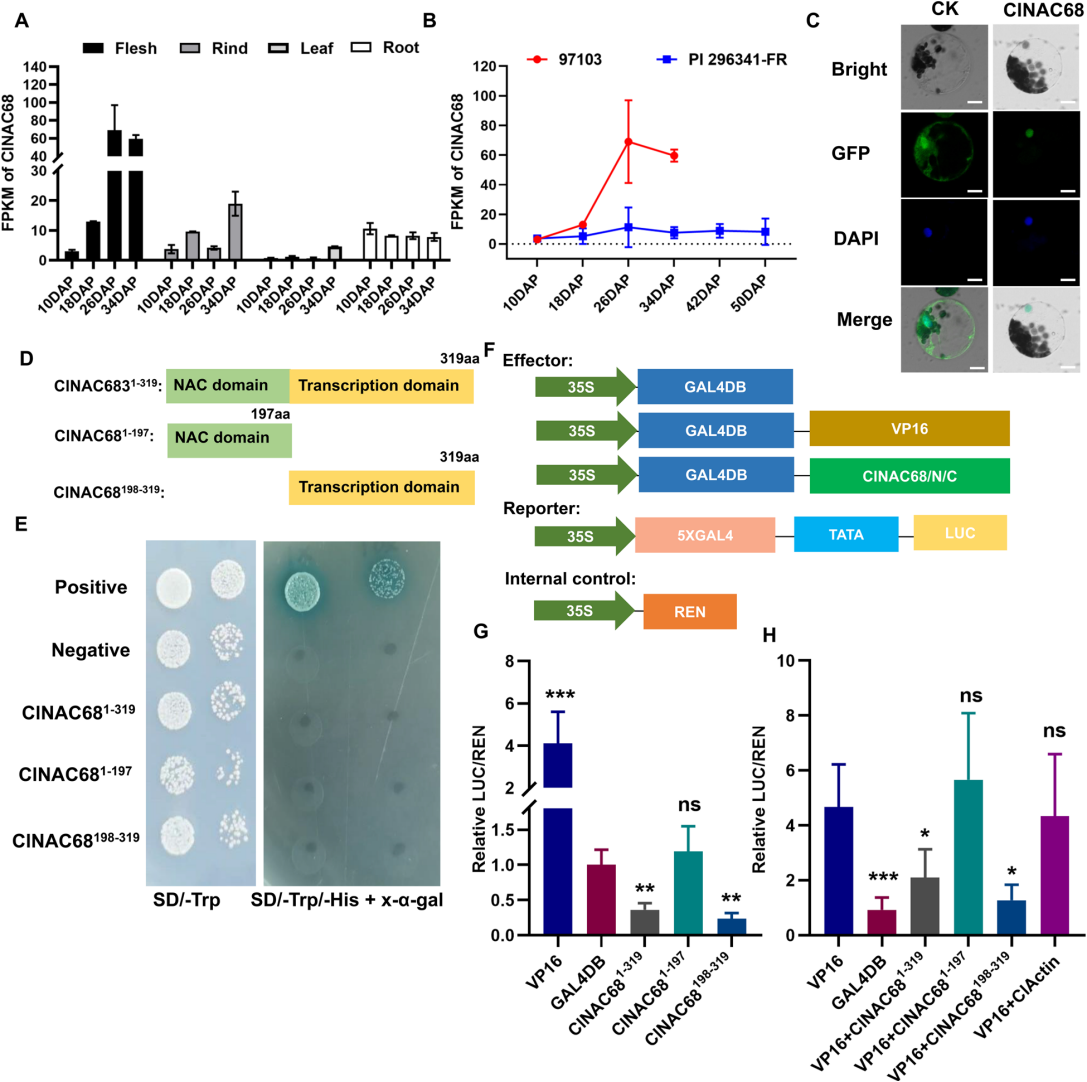
ClNAC68 is highly expressed in flesh and is a transcriptional repressor. **A**. The expression pattern of *ClNAC68* in different tissues. DAP days after pollination, FPKM Fragments Per Kilobase per Million mapped reads. **B** The expression pattern of *ClNAC68* in cultivated and wild watermelons during fruit ripening. **C**. *ClNAC68* was localized in the nucleus. pYBA1332 was used as the negative control and is indicated as CK. Bar = 10 μm. **D** Schematic representation of different lengths of *ClNAC68*. Full-length *ClNAC68*: *ClNAC68*^1–319^, N-terminus of *ClNAC68*: *ClNAC68*^1–197^, and C-terminus of *ClNAC68*: *ClNAC68*^198–319^. **E** Growth performance of yeast cells containing *ClNAC68*^1–319^, *ClNAC68*^1–197^, and *ClNAC68*^198–319^ on SD-T medium and SD-TH medium containing X-α-gal. pGBKT7-P53 and pGBKT7 were used as the positive and negative controls, respectively. **F** Schematic representation of different effectors, reporters, and internal controls. **G** Transcriptional regulatory activity of *ClNAC68*. The GALDB-VP16 and GALDB vectors were used as the positive and negative controls, respectively. Five independent replicates were performed. The asterisks denote significance compared with the negative control by two-way ANOVA: *p *<* 0.05, **p < 0.01. **H** Effects of different lengths of *ClNAC68* on VP16-mediated LUC gene expression. The GALDB-VP16 and GALDB vectors were used as the positive and negative controls, respectively. Watermelon ClActin was used as the noninteractive control. Five independent replicates were performed. The asterisks denote significance compared with VP16 by two-way ANOVA: *p < 0.05, **p < 0.01, ***p < 0.001

A previous study showed that there are 80 NAC transcription factors encoded in the watermelon genome and that 21 *ClNACs* are expressed in both the vascular tissues and fruit^22^. Among these NAC genes, *ClNAC68* (Cla97C03G059250, Cla019693 in version 1 of the watermelon genome) was highly expressed at 18, 26, and 34 DAP, and its expression was significantly higher in flesh than in rind. We further tested the expression of *ClNAC68* in different tissues and found that the expression of ClNAC68 was highest in flesh and lowest in leaves (Fig. 1A) and was higher in cultivated watermelon (97103) than in wild watermelon (PI296341-FR) (Fig. 1B). The *ClNAC68*-GFP fusion protein was detected by confocal microscopy and was observed to be localized in the nucleus (Fig. 1C).

To test the transcriptional activity of *ClNAC68*, we generated N- and C-terminal *ClNAC68* constructs containing the NAC domain (aa 1–197) and transcription domain (aa 198–319), respectively (Fig. 1D). Then, Y2H and dual-luciferase reporter assays were performed. For the Y2H assay, all yeast cells containing BD-*ClNAC68*^1–319^, BD-*ClNAC68*^1–197^, BD-*ClNAC68* ^198–319^, or the positive or negative control survived in SD-T medium, while only those containing the positive control survived and appeared blue in SD-Trp/-His medium containing X-α-gal. No other yeast cells survived (Fig. 1E). These results indicated that *ClNAC68* had no transcriptional activation activity. Then, we performed a dual-luciferase reporter assay to further detect its transcriptional activity in watermelon protoplasts. Plasmids were constructed as shown in Fig. 1F. The relative luciferase activities of full-length *ClNAC68*^1–319^ and the transcription domain *ClNAC68*^198–319^ were significantly lower than those of the negative control, while there was no significant difference in relative luciferase activity between the NAC domain *ClNAC68*^1–197^ and the negative control (Fig. 1G). Furthermore, these three constructs were individually coexpressed with VP16, and both the full-length *ClNAC68*^1–319^ and the transcription domain *ClNAC68*^198–319^ had significant inhibitory effects on VP16-induced gene expression, while the NAC domain *ClNAC68*^1–197^ and the negative control had no inhibitory effects (Fig. 1H). These results indicated that ClNAC68 is a transcriptional repressor and that its transcriptional repression domain is located in the C-terminus.

### Knockout of *ClNAC68* decreased sugar accumulation in flesh

**Fig. 2:**
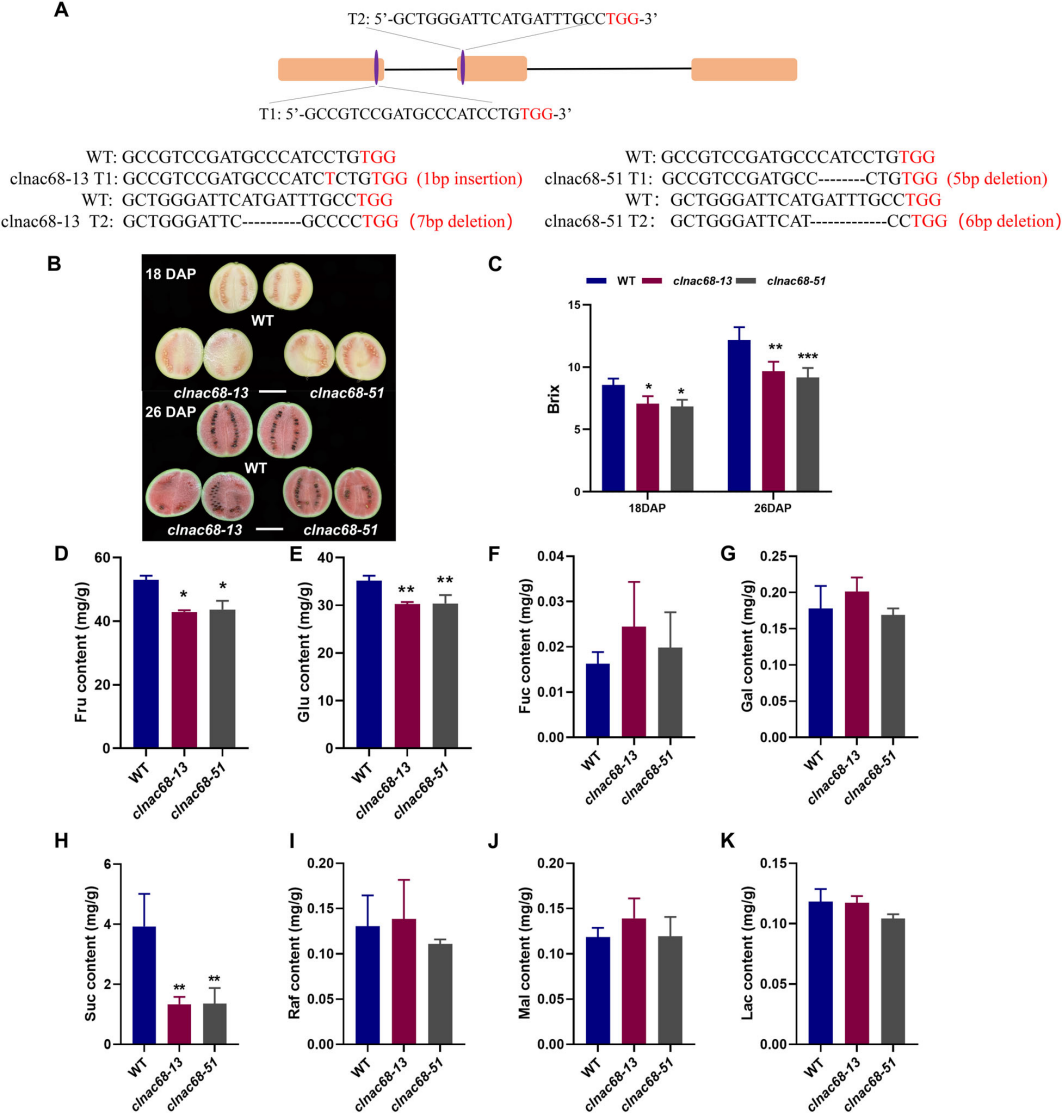
Knockout of ClNAC68 decreased sugar accumulation. **A** Gene editing of *clnac68*-13 and *clnac68*-51. **B** Phenotype of WT and *clnac68* mutant fruits at 18 DAP and 26 DAP. Bar = 10 cm. **C** Brix of WT and *clnac68* mutant fruits at 18 DAP and 26 DAP. **D–K** Fructose **D**, glucose **E**, fucose **F**, galactose **G**, sucrose **H**, raffinose **I**, maltose **J**, and lactose **K** contents in WT and *clnac68* mutant fruits at 26 DAP. The asterisks denote significance compared with WT fruits at different developmental stages by two-way ANOVA: *p < 0.05, **p < 0.01, ***p < 0.001

To explore how *ClNAC68* regulates fruit ripening and quality, we knocked out *ClNAC68* by the CRISPR-Cas9 system (*clnac68*) in the cultivated inbred watermelon line ZZJM. The *clnac68-13* mutant line showed a 1 bp insertion in the first target site and a 7 bp deletion in the second target site. The *clnac68-51* mutant line showed a 5 bp deletion in the first target site and a 6 bp deletion in the second target site (Fig. 2A). Both the mutant lines exhibited loss of function of *ClNAC68*.

There was no significant difference in plant growth performance between the WT and *clnac68* lines during plant development and fruit ripening. At 18 DAP and 26 DAP, the flesh color showed no significant difference between the WT fruits and *clnac68* mutant fruits (Fig. 2B). The brix of *clnac68* mutant fruits was significantly lower than that of WT fruits at both 18 DAP and 26 DAP, but the difference in brix at 18 DAP was less significant than that at 26 DAP (Fig. 2C). GC/MS analysis showed that among fructose, glucose, fucose, galactose, sucrose, raffinose, maltose, and lactose, only fructose, glucose, and sucrose exhibited a significantly lower content in both the *clnac68*-13 and *clnac68*-51 mutant flesh than in the corresponding WT flesh at 26 DAP (Fig. 2D-K). These results indicated that *ClNAC68* positively regulated the accumulation of sugars, especially sucrose, fructose, and glucose, at the mature fruit ripening stage.

A previous study proved that IAA and ABA are important hormones that positively regulate fruit ripening and sugar accumulation^26,27^. Therefore, we measured the contents of free IAA and ABA in flesh at 18 DAP and 26 DAP, respectively. Free IAA and ABA were significantly lower at both the 18 DAP and 26 DAP in *clnac68* mutant fruit than in WT fruit, and the difference in free IAA and ABA at 26 DAP was more significant than the difference at 18 DAP (Figure S1A and B). These results indicated that *ClNAC68* might regulate watermelon fruit sweetness and ripening by mediating the contents of free IAA and ABA.

### Knockout of *ClNAC68* delayed seed maturation and inhibited seed germination and root elongation

**Fig. 3:**
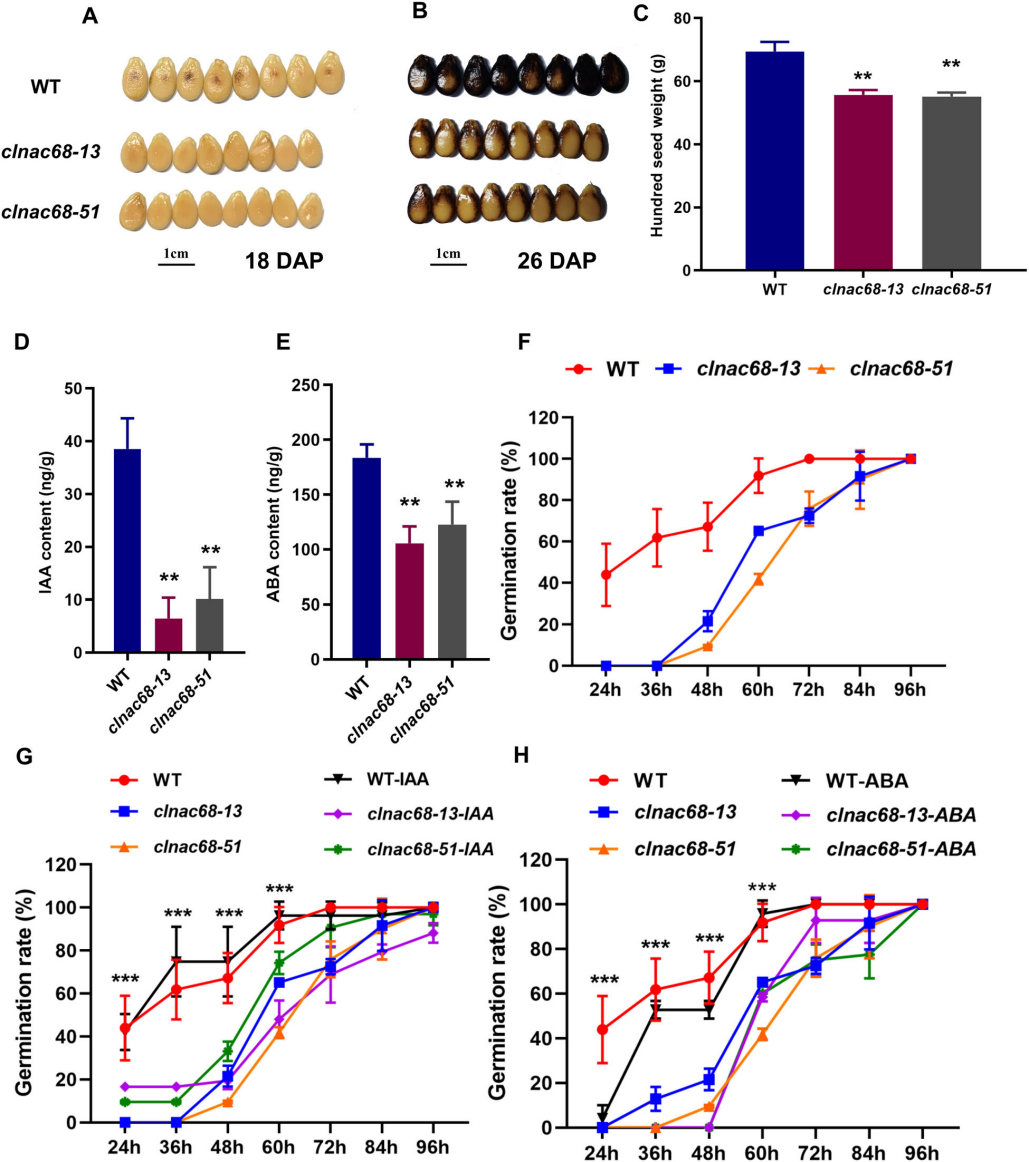
Knockout of *ClNAC68* inhibited seed germination. **A–B** Phenotype of WT and *clnac68* mutant seeds at 18 DAP **A** and 26 DAP **B.** Bar=1 cm. **C** Hundred-seed weight of WT and *clnac68* mutant lines. **D–E** Free IAA **D** and ABA **E** contents in WT and *clnac68* mutant seeds at 26 DAP. **F** Seed germination rate of WT and *clnac68* mutant lines under normal conditions. **G–H**. Seed germination rate of WT and *clnac68* mutant lines under treatment with 10 μM IAA **G** or 10 μM ABA **H**. The asterisks denote significance compared with WT plants at different developmental stages by two-way ANOVA: *p < 0.05, **p < 0.01, ***p < 0.001

Although there no significant difference in fruit ripening was found between the WT and *clnac68* mutant fruits, the maturation of *clnac68* mutant seeds was obviously delayed. At 18 DAP, the seeds of the WT line started to undergo coloration, while those of the *clnac68* mutant lines remained yellow (Fig. 3A). At 26 DAP, the seed color of WT lines became completely black, while *clnac68* mutant seeds were unevenly colored (Fig. 3B). The length of mutant seeds was also shorter than that of WT seeds (Figure S2A), while the seed width was not significantly different (Figure S2B). The hundred-seed weight of *clnac68* mutant seeds was significantly lower than that of WT seeds (Fig. 3C). Due to the significantly different seed phenotype between the WT and *clnac68* mutant lines, we measured hormone levels in seeds of WT and *clnac68* mutant lines at 26 DAP and found that the IAA and ABA contents were also significantly lower in *clnac68* mutant seeds (Fig. 3D and E). Then, we further tested the seed germination rate and root elongation performance. After 72 h of germination, WT seeds had completely germinated, while the germination rate of *clnac68* mutant seeds was significantly lower, only 60%. *clnac68* mutant seeds had completely germinated after 96 h of germination (Fig. 3F). To further confirm the effect of IAA and ABA on mutant seed germination, we measured the seed germination rates of the WT and *clnac68* mutant lines after treatment with exogenous IAA and ABA. The seed germination rate of the *clnac68* lines after exogenous IAA treatment was significantly higher than that after control (H_2_O) treatment, which indicated that IAA could partially restore seed germination in the *clnac68* mutant (Fig. 3G). However, ABA treatment delayed seed germination in both the WT and *clnac68* lines, and *clnac68* mutant seeds started germinating after 60 h and germinated much more slowly than WT seeds (Fig. 3H).

**Fig. 4:**
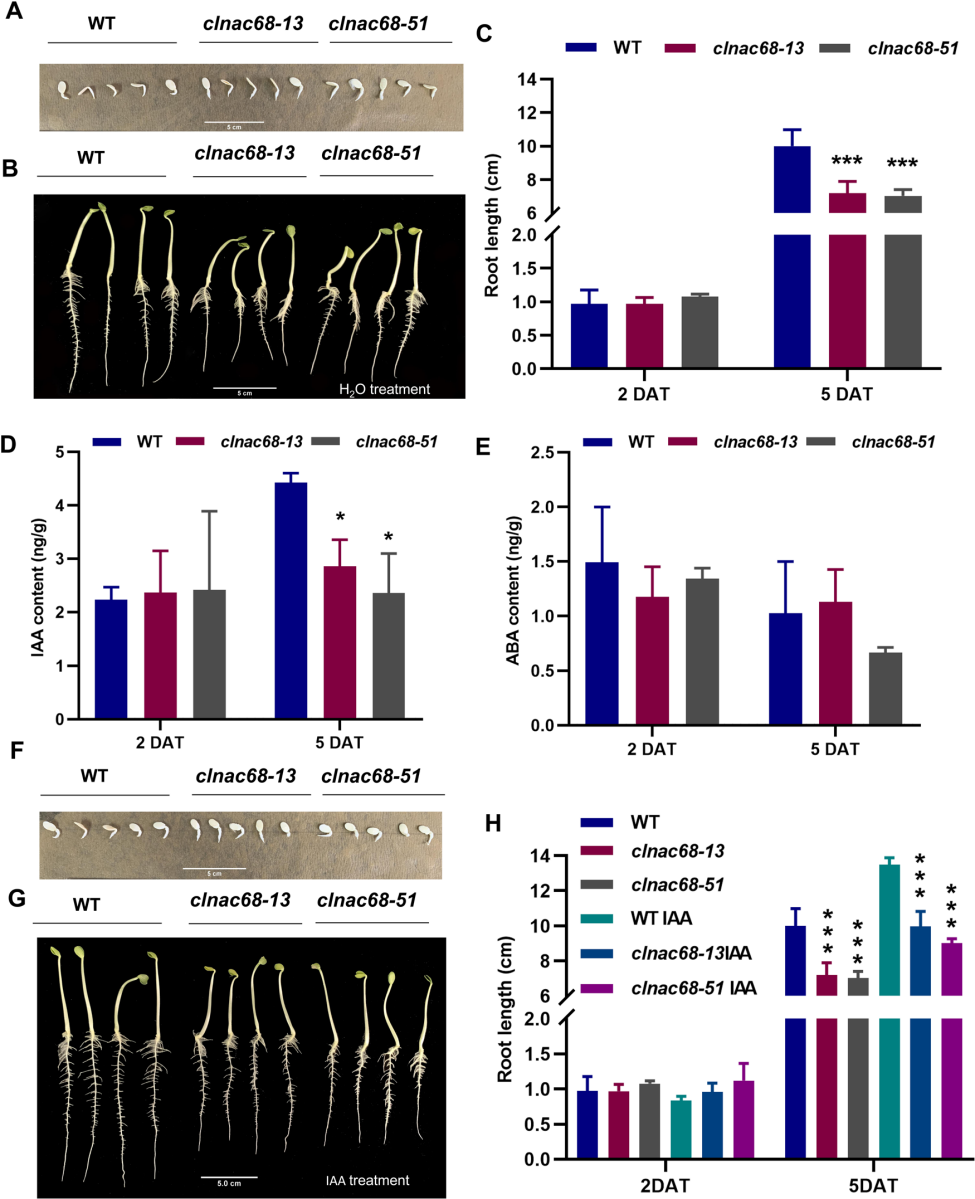
Knockout of *ClNAC68* inhibited root elongation. **A–B** Phenotype of WT and *clnac68* mutant root elongation at two DAT **A** and five DAT **B.** **C** Root length (cm) of the WT and *clnac68* mutant lines at two DAT and five DAT. **D–E**. Free IAA **D** and ABA **E** contents in roots in the WT and *clnac68* mutant lines at two DAT and five DAT. **F–G** Phenotype of WT and *clnac68* mutant root elongation under treatment with 10 μM IAA at two DAT **F** and five DAT **G**. **H** Root length (cm) of the WT and *clnac68* mutant lines at two DAT and five DAT under H_2_O or IAA treatment. The asterisks denote significance compared with WT plants at different developmental stages by two-way ANOVA: *p < 0.05, **p < 0.01, ***p < 0.001

Regarding root elongation, the comparison between the WT and *clnac68* mutant lines showed that after 2 days of treatment, there was no significant difference in root length between the WT line and *clnac68* mutant lines (Fig. 4A). After 5 days of treatment, the root length in the *clnac68* mutant lines was significantly lower than that in the WT line (Fig. 4B and C). Furthermore, the free IAA content in roots of the *clnac68* mutant lines was significantly lower than that in the WT line (Fig. 4D) but the ABA content showed no significant difference between the WT and *clnac68* mutant lines (Fig. 4E). Then, we further evaluated root elongation under treatment with exogenous free IAA. The root length of both the WT and *clnac68* mutant lines was significantly increased after treatment with exogenous free IAA but the root length of the *clnac68* mutant lines was significantly shorter than that of the WT line (Fig. 4F-H). These results indicated that *ClNAC68* positively regulates seed germination and root elongation via the increased free IAA content.

### ClNAC68 binds directly to the invertase gene *ClINV* and represses its expression

To further explore the regulatory mechanism of *ClNAC68*, transcriptome analysis was performed on flesh and seeds at 26 DAP, which showed more significant differences in the *clnac68* mutant. The ripening-related differentially expressed genes (DEGs) (*p-*adjust < 0.05 and | log2FC | >= 1) between the WT and *clnac68-*13 lines were selected to explore the potential target gene of *ClNAC68*. There were 501 DEGs (195 upregulated and 306 downregulated) at 26 DAP in *clnac68*-13 mutant fruits (Figure S3A), and there were 958 DEGs (527 upregulated and 431 downregulated) at 26 DAP in seeds (Figure S3B). Of these DEGs, 83 were found in both the fruits and seeds at 26 DAP, and 835 DEGs and 418 DEGs were specifically expressed in seeds and flesh, respectively, at 26 DAP (Figure S3C). KEGG analysis showed that the DEGs in flesh were clustered mainly into the energy metabolism, carbohydrate metabolism, and signal transduction pathways, while the DEGs in seeds were mainly clustered into the amino acid metabolism, biosynthesis of other secondary metabolites, carbohydrate metabolism, and signal transduction pathways (Figure S3D and E).

**Fig. 5:**
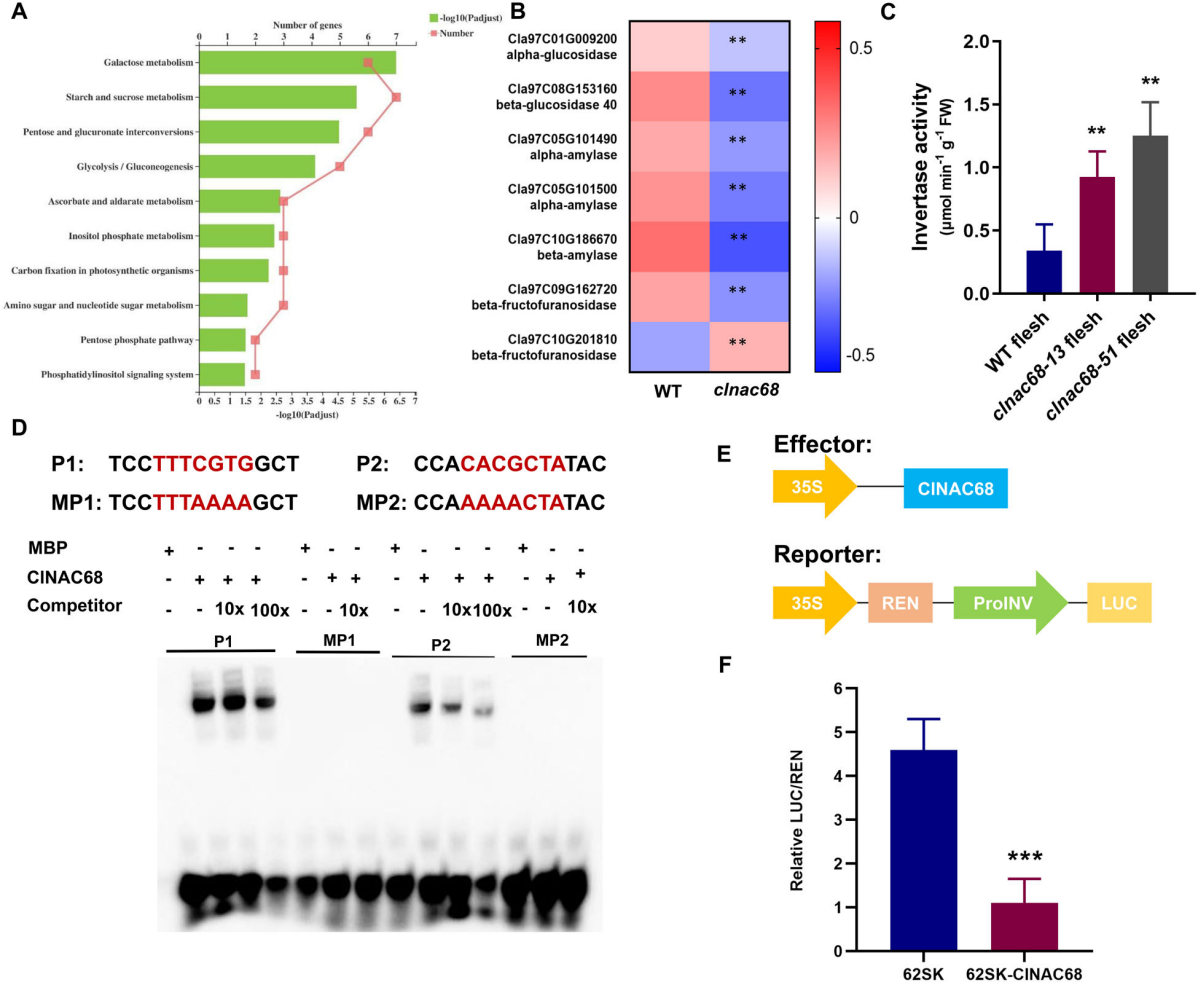
ClNAC68 directly binds to ClINV and represses its expression. **A** KEGG enrichment analysis of DEGs involved in carbohydrate metabolism. **B** Heatmap analysis of DEGs involved in starch and sucrose metabolism in WT and *clnac68* mutant fruits. **C** Invertase activity in WT and *clnac68* mutant fruits at 26 DAP. The error bars show the standard deviations of three independent replicates. **D** EMSAs showed that ClNAC68 bound to the *ClINV* promoter. P1 and P2 denote the two different binding sites. MP1 and MP2 denote binding of the mutant probe to P1 and P2. Competitor denotes non-biotin-labeled probes. The untagged MBP protein incubated with biotin-labeled probes was used as the negative control. **E**. Schematic representation of effector and reporter vectors. **F** The transient dual-luciferase assay showed that *ClNAC68* significantly repressed *ClINV* expression. Five independent replicates were performed. The asterisks denote significance compared with the negative control by two-way ANOVA: *p < 0.05, **p < 0.01, ***p < 0.001

KEGG enrichment analysis was used to further investigate the DEGs in carbohydrate metabolism, including those related to sugar, coloration, metabolism, and hormone biosynthesis, in flesh. The top three enriched pathways were ‘galactose metabolism’, ‘starch and sucrose metabolism,’ and ‘pentose and glucuronate interconversions’ (Fig. 5A). These results indicated that *ClNAC68* participated in sugar metabolism regulation in flesh. There were 7 DEGs involved in starch and sucrose metabolism: two invertase genes (beta-fructofuranosidase) involved in regulating sucrose degradation^5^, three amylase genes, an alpha-glucosidase involved in regulating hydrolysis of polysaccharides^28^, and a beta-glucosidase involved in regulating the free ABA content by catalyzing the conjugation of Glc to ABA (to form ABA-GE)^29,30^. Heatmap analysis of these DEGs showed that only one invertase gene, Cla97C10G201810, was significantly upregulated in *clnac68* mutant fruits and that all the other genes were significantly downregulated in *clnac68* mutant fruits (Fig. 5B). Cla97C10G201810 belongs to the cell wall invertase family, and we found that cell wall invertase activity was significantly higher in *clnac68* mutant fruits (Fig. 5C). These results were consistent with the lower sucrose content in *clnac68* mutant fruits. Furthermore, *ClNAC68* was found to be a transcriptional repressor, and *ClNAC68* might mediate the upregulation of Cla97C10G201810 (an invertase gene) expression and invertase activity to control sugar accumulation.

To test this hypothesis, EMSA and dual-luciferase assays were performed. The motifs (T/A)(T/G)(A/G/C)CGT(G/A)(T/A) were previously reported as NAC transcription factor binding sites^31^. We analyzed the promoter (2 kb upstream of the start codon) of *ClINV*, and two binding sites were found. The EMSA showed that *ClNAC68* bound to all the cis-elements in *ClINV* and that the level of bound complex decreased as the concentration of competitors increased, while ClNAC68 could not bind to the mutant probes. MBP could not bind to these probes (Fig. 5D). In the transient dual-luciferase assay, pGreenII 62-SK-*ClNAC68* was used as the effector, and proClINV-LUC was used as the reporter (Fig. 5E). The LUC/REN ratio in leaves coexpressing *ClNAC68* and proClINV-LUC was significantly lower than the LUC/REN ratio in mock control leaves (Fig. 5F). These results indicated that ClNAC68 directly binds to the promoter of *ClINV* and represses its expression.

Sugars Will Eventually be Exported Transporters (SWEET) were identified as novel sugar transporters and were characterized as candidate genes for maintaining sugar homeostasis in sink tissues. In this study, we analyzed *SWEET* gene expression in *clnac68* mutant fruits. Nine *SWEET* genes were found, and three *SWEET*s were significantly downregulated in the *clnac68* mutant lines (Figure S4).

### ClNAC68 directly binds to the IAA deactivator *ClGH3.6* and represses its expression

In this study, we found that the free IAA and ABA contents in both the flesh and seeds were significantly lower in *clnac68* mutant lines. Thus, we focused on the DEGs in the related hormone biosynthesis and signaling pathways. KEGG enrichment analysis showed eight DEGs involved in the metabolism of terpenoids and polyketides; among these, one and three DEGs in flesh and seeds, respectively, were clustered into carotenoid biosynthesis (including ABA biosynthesis); two and five DEGs were clustered into diterpenoid biosynthesis (including gibberellin biosynthesis); and three and one DEGs were clustered into brassinosteroid biosynthesis. However, no DEGs were clustered into tryptophan metabolism (including IAA biosynthesis) in either flesh or seeds. Due to the reductions in free IAA and ABA, we analyzed the DEGs involved in ABA biosynthesis, and we found that the expression of the key ABA biosynthesis gene Cla97C07G137260, encoding 9-cis-epoxycarotenoid dioxygenase (NCED), was significantly lower in the flesh of *clnac68* mutant fruits than in that of WT fruits at 26 DAP (Figure S5A). In seeds, the expression of ABA biosynthesis genes, including the violaxanthin de-epoxidase (*VDE*) gene Cla97C11G216330, *NCED* gene Cla97C06G117340, and ABA catabolic gene *CYP707A* Cla97C07G137840, was significantly lower in the *clnac68* mutant lines (Figure S5B). Given that *ClNAC68* was identified as a transcriptional repressor, these lower expression levels indicated that the DEGs involved in ABA metabolism are not the target genes of *ClNAC68*.

**Fig. 6:**
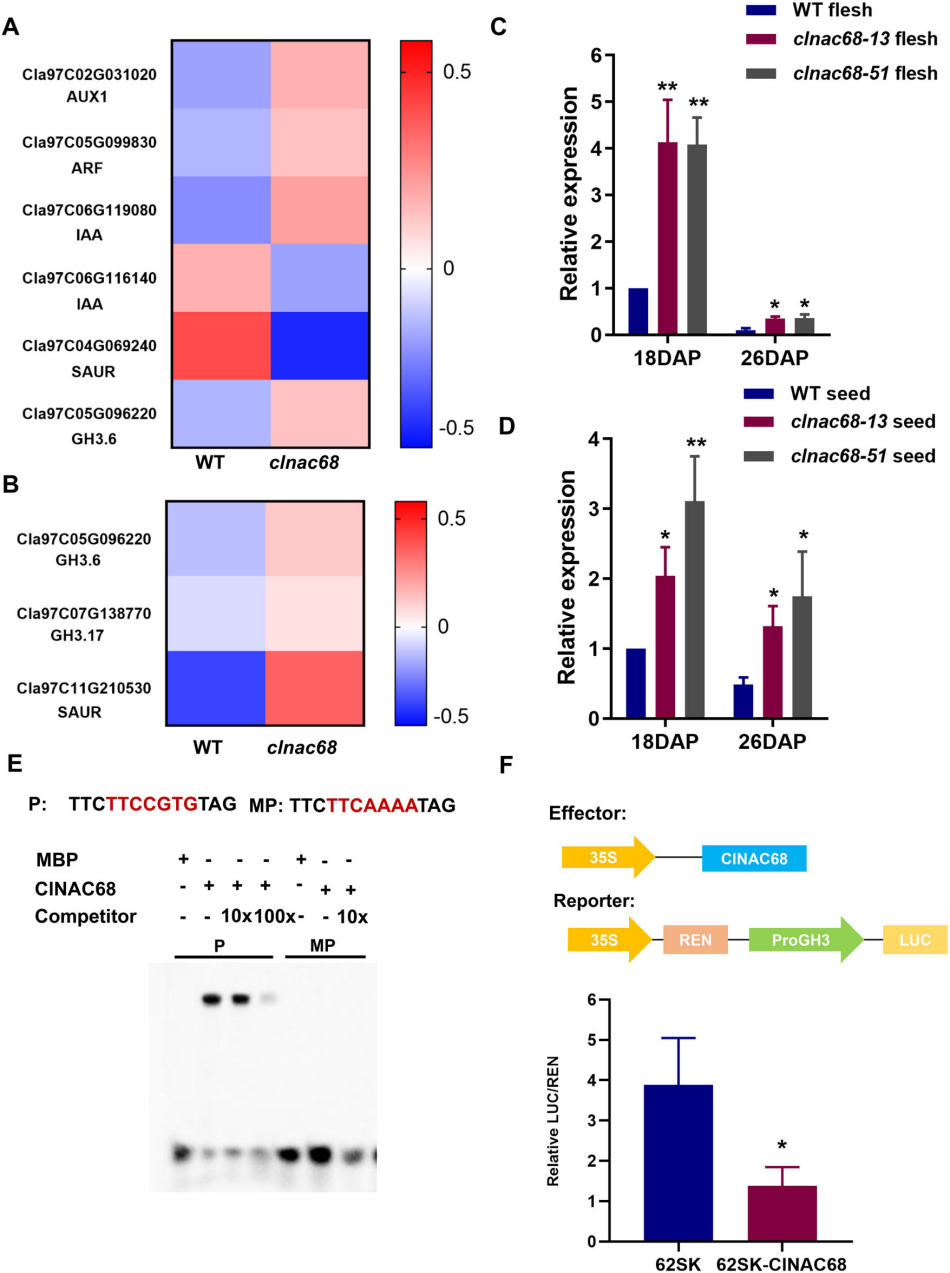
ClNAC68 directly binds to ClGH3.6 and represses its expression. **A–B** Heatmap of DEGs in the IAA signaling pathway in WT and *ClNAC68* fruits **A** and seeds **B**. **C–D**. Relative expression of *ClGH3.6* in WT and *clnac68* mutant fruits **C** and seeds **D** at 18 and 26 DAP. *ClActin* was used as the internal control. The error bars show the standard deviations of three independent replicates. **E** EMSAs showed that *ClNAC68* bound to the *ClGH3.6* promoter. MP denotes the mutant probe, and Competitor denotes the nonbiotin-labeled probes. The untagged MBP protein incubated with biotin-labeled probes was used as the negative control. **F** The transient dual-luciferase assay showed that *ClNAC68* significantly repressed *ClGH3.6* expression. Five independent replicates were performed. Empty pGreen62SK vector was used as the negative control. The asterisks denote significance compared with the negative control by two-way ANOVA: *p < 0.05, **p < 0.01, ***p < 0.001

Given that no DEGs were involved in IAA biosynthesis, we further analyzed the plant hormone signaling pathways in both the flesh and seeds to understand how ClNAC68 regulates the content of free IAA. The DEGs in flesh and seeds were clustered mainly into the IAA signaling pathway. In flesh, four DEGs were upregulated, including auxin transporter (*AUX1*), auxin response factor (*ARF*), GH3, and auxin-responsive protein (*AUX/IAA*), while a gene encoding an auxin-responsive family protein (*SAUR*) was downregulated (Fig. 6A). In seeds, two *GH3* genes and a *SAUR* gene were upregulated in *clnac68* mutant seeds (Fig. 6B). Among these DEGs, only Cla97C05G096220, encoding *GH3.6*, was upregulated in both seeds and flesh. GH3.6 is a deactivator of IAA, and the expression of *GH3.6* showed a negative correlation with the free IAA content^32^. Therefore, we predicted that *ClNAC68* might regulate the free IAA content by repressing *GH3.6* expression.

To assess the relationships between *ClNAC68* and IAA signaling genes, we analyzed the promoters of these upregulated genes and found a NACRS in the promoter of *ClGH3.6* but not in the other genes. The relative expression of *ClGH3.6* was significantly higher in both the flesh and seeds at 18 DAP and 26 DAP (Fig. 6C and D). EMSA showed that ClNAC68 could bind to the NACRS in the *ClGH3.6* promoter and that the binding activity decreased with an increase in the probe concentration, while ClNAC68 could not bind to the mutant probe, and the negative control could not bind to the NACRS (Fig. 6E). Further transient dual-luciferase assays were performed in tobacco, and the results showed that in the presence of *ClNAC68*, the LUC/REN ratio was significantly lower than the LUC/REN ratio in the mock control (Fig. 6F). These results indicated that *ClNAC68* can directly bind to the promoter of *ClGH3.6* and suppress its expression. In summary, these results demonstrated that *ClNAC68* increases the accumulation of free IAA in both the flesh and seeds by directly repressing the expression of the IAA deactivator *ClGH3.6*.

## Discussion

NAC is one of the largest gene families in plants and is involved in stress tolerance regulation^33^, plant development, and fruit ripening^16,34^. In banana, *MaNAC1* and *MaNAC2* are transcriptional repressors and negatively regulate *MaERF11* to induce ethylene accumulation and fruit ripening^35^. Most previous studies focused on mechanisms of development and ripening, and studies of how NAC transcription factors regulate fruit quality are limited. In maize, *ZmNAC34* functions as a transcriptional repressor, and overexpression of *ZmNAC34* decreases the soluble solid content and starch content by downregulating the expression of starch biosynthesis-related genes^36^. In this study, we characterized the transcriptional repressor *ClNAC68*, which was highly expressed in sweet flesh (Fig. 1). Knockout of *ClNAC68* resulted in reduced sugar accumulation, and the total contents of sugar, sucrose, fructose, and glucose were significantly lower in *clnac68* mutant fruits, especially at 26 DAP (Fig. 2). Cla97C10G201810, belonging to the cell wall invertase family, is directly regulated by *ClNAC68* (Fig. 5). A previous study showed that silencing lycopersicum invertase 5 (*LIN5*), a cell wall invertase in tomato, reduced brix and the fructose content but increased sucrose accumulation^37^. In *clnac68* mutant fruits, ClINV activity was increased, and this increase might induce sucrose degradation and result in a decrease in the total sugar content and sucrose accumulation. These results indicated that *ClNAC68* regulates the sugar content by repressing invertase activity in watermelon. Sucrose is transported from photosynthetic leaf sources to nonphotosynthetic sink organs by sucrose transporters (SUTs) and is then hydrolyzed by invertases and sucrose synthases to maintain sink strength^38^. Significant changes in the metabolic enzymes involved in sugar metabolism directly affect sweetness in fruits^39^, and sugar transporters control sucrose allocation^40^. SWEET, as a sugar transporter, directly determines the sugar content in fruits^41^. In this study, three *SWEET*s were significantly downregulated in the *clnac68* mutant lines. Lower sweet activity might result in lower glucose and fructose contents (Figure S4).

Plant hormones play important roles in the regulation of plant growth, development, and fruit ripening^2^. In our previous study, we found that the ABA content increased during watermelon fruit ripening, and that treatment with exogenous ABA or an ABA inhibitor could induce or inhibit fruit ripening, respectively^26^. The ABA content was significantly lower in *clnac68* mutant fruits, and transcriptome data showed that the expression of the ABA biosynthesis gene *NCED* (Cla97C07G137260) was significantly lower in mutant fruits (Figure S5A). Moreover, Cla97C08G153160 encodes a beta-glucosidase and is homologous to *AtBG1*, which catalyzes the formation of ABA-GE to activate ABA and was significantly downregulated in mutant fruits (Figure S5A). These results indicated that *ClNAC68* may affect the ABA content by downregulating *ClNCED* and *ClBG1*. An increasing number of studies have shown that NAC transcription factor can directly bind to the ABA biosynthesis gene *NCED* to regulate ABA accumulation. Given that ClNAC68 was found to be a transcriptional repressor, the downregulated *ClNCED* and *ClBG1* were not its direct targets in this study. More comprehensive research is needed to explore the interaction mechanism between ClNAC68 and other regulators of ABA biosynthesis in the future.

The fruit ripening process is regulated by the metabolism of multiple hormones and by multiple signaling pathways. In contrast to the positive regulatory function of ABA in fruit ripening, the function of IAA in the regulation of fruit ripening is still under debate. Previous studies showed that the IAA content decreased during fruit ripening and affected fruit acidity and sugar accumulation^42,43^, while several studies showed that the IAA content increased during fruit ripening and peaked at the mature stage in peach^27^. In this study, we found that the content of free IAA increased during fruit ripening and seed maturation and was significantly lower in both the fruits and seeds of the *clnac68* mutants (Figs. 3 and 4). Transcriptome analysis showed several DEGs in the IAA signaling pathway, but the function of IAA signaling in the regulation of fruit ripening is still unclear. *VcIAA27* plays a negative role in the IAA signaling pathway^44^, while *PpIAA19* expression increases during fruit ripening to change fruit shape^45^. The other key component, ARF, also showed opposite functions during fruit ripening: overexpression of *SlARF10* accelerated fruit ripening and induced sugar accumulation^46^; however, overexpression of *SlARF4* delayed fruit ripening^42^. Both the Cla97C06G119080, encoding Aux/IAA, and Cla97C05G099830, encoding ARF, were significantly upregulated in *clnac68* mutant fruits, but how they regulate watermelon fruit ripening is still unknown. Further studies are needed to confirm the functions of IAA and IAA signaling in the regulation of watermelon fruit ripening.

Seed development and seed size are also affected by the levels of multiple hormones. In a previous study, the free IAA content was found to be increased during development in both the variant with larger seeds and the variant with smaller seeds, and the free IAA content was significantly higher in the variant with larger seeds^14^. In this study, seed maturation was delayed and the seed size was decreased in the *clnac68* mutants. We suspected that the lower free IAA content in *clnac68* mutant seeds might result in this phenotype. Due to delayed seed maturation, the germination and root elongation of *clnac68* mutant seeds were significantly decreased compared with those of WT seeds. The expression of two GH3s (Cla97C05G096220 and Cla97C07G138770) involved in IAA signaling was increased in mutant seeds, while no DEGs involved in IAA biosynthesis were identified in either flesh or seeds. Further EMSA and transient dual-luciferase assays showed that ClNAC68 can directly bind to the NACRS in the promoter of *ClGH3.6* (Fig. 6). These results indicated that *ClNAC68* mediates the negative regulation of the IAA deactivator *ClGH3.6* to increase the free IAA content in seeds, and these results were supported by a previous study^20^.

ABA induces seed dormancy and inhibits seed germination^47^, and we found that the ABA content was significantly lower in *clnac68* mutant seeds than in WT seeds. The DEGs in the ABA biosynthesis pathway, including *NCED* and *VDE*, were significantly downregulated in the *clnac68* mutant, while *CYP707A*, involved in ABA catabolism, was also significantly downregulated in mutant seeds. Given that *ClNAC68* was found to be a transcriptional repressor, these downregulated DEGs were not the target of *ClNAC68*. A previous study showed that this NAC transcription factor positively regulates *ABI5* expression to increase ABA sensitivity and affect seed germination^48^. In this study, neither WT nor *clnac68* mutant seeds showed hypersensitivity to exogenous ABA treatment, and no DEGs in the ABA signaling pathway were identified in *clnac68* mutant seeds. These results might indicate that ClNAC68 mainly regulates seed development via the IAA signaling pathway and then affects seed germination and root elongation. How NAC regulates ABA biosynthesis and signaling in watermelon is still unknown, and more research is needed to explore the regulatory mechanism in both flesh and seeds.

In summary, *ClNAC68* was found to be a transcriptional repressor that was highly expressed in flesh. Knockout of *ClNAC68* significantly reduced the sucrose, fructose, and glucose contents. ClNAC68 directly inhibited *ClINV* expression and invertase activity to prevent sucrose degradation. Furthermore, ClNAC68 might regulate the free IAA content by directly suppressing *ClGH3.6* expression in seeds, resulting in seed maturation and germination. These findings revealed that ClNAC68 plays an important role in sugar metabolism and the IAA signaling pathway and provide new insight for modifying breeding practices to improve the fruit quality of watermelon.

## Supplementary information

supplemental information
