# Correction: The NAC transcription factor *ClNAC68* positively regulates sugar content and seed development in watermelon by repressing *ClINV* and *ClGH3.6*

**DOI:** 10.1038/s41438-021-00710-z

**Published:** 2021-12-18

**Authors:** Jinfang Wang, Yanping Wang, Jie Zhang, Yi Ren, Maoying Li, Shaowei Tian, Yongtao Yu, Yi Zuo, Guoyi Gong, Haiying Zhang, Shaogui Guo, Yong Xu

**Affiliations:** National Watermelon and Melon Improvement Center, Beijing Academy of Agricultural and Forestry Sciences, Key Laboratory of Biology and Genetic Improvement of Horticultural Crops (North China), Beijing Key Laboratory of Vegetable Germplasm Improvement, Beijing, 100097 China

Correction to: *Horticulture Research* **8**:214

10.1038/s41438-021-00649-1 published online 01 October 2021

In this article^1^, author name Yanping Wang was incorrectly tagged as corresponding author. Prof. Wang should be the co-first author, instead of the corresponding author.

The original article has been corrected.
