# Supplementary material for: The NAC transcription factor ClNAC68 positively regulates sugar content and seed development in watermelon by repressing ClINV and ClGH3.6
Source: Hortic Res. 2021 Oct 1;8:214. doi: 10.1038/s41438-021-00649-1 (PMC8484586; doi:10.1038/s41438-021-00649-1)
Supplement: Supplementary file 1 — supplemental information [file 41438_2021_649_MOESM1_ESM.docx]

**The NAC transcription factor *ClNAC68* positively regulates watermelon sugar content and seed development by repressing *ClINV* and *ClGH3.6***

Jinfang Wang ^a^ ^#^, Yanping Wang ^a^ ^#^, Jie Zhang ^a^, Yi Ren ^a^, Maoying Li ^a^, Shaowei Tian ^a^, Yongtao Yu ^a^, Zuo Yi ^a^, Guoyi Gong ^a^, Haiying Zhang ^a^ *, Shaogui Guo ^a^ *, Yong Xu ^a^

^a^ National Watermelon and Melon Improvement Center, Beijing Academy of Agricultural and Forestry Sciences, Key Laboratory of Biology and Genetic Improvement of Horticultural Crops (North China), Beijing Key Laboratory of Vegetable Germplasm Improvement, Beijing, 100097, China

#These authors contribute equally to this work.

Table S1. The primer list used in this study.

| Purpose | Gene | Primers |
| --- | --- | --- |
| ORF of ClNAC68  gene | ClNAC68  Cla97C03G059250 | F: ATGACTTGGTGCAGTGGCTCG  R: TCAGATTTTCCTGTCGAGCTTTC |
| Subcellular location  (pYBA1332) | ClNAC68 | F:cgctctagaactagtggatccATGACTTGGTGCAGTGGCTCG  R:gataagcttgatatcgaattcGATTTTCCTGTCGAGCTTTC |
| Transcription activation in yeast  (pGBKT7) | ClNAC68^1-319^  ClNAC68^1-197^  ClNAC68^198-319^ | F:atggccatggaggccgaattcATGACTTGGTGCAGTGGCTCG  R:ccgctgcaggtcgacggatccTCAGATTTTCCTGTCGAGCTTTC  F:atggccatggaggccgaattcATGACTTGGTGCAGTGGCTCG  R:ccgctgcaggtcgacggatccGAGGTGGTATTGGTGCATGACC  F:atggccatggaggccgaattcGGCAACAACGAGGAAGAGAAAG  R:ccgctgcaggtcgacggatccTCAGATTTTCCTGTCGAGCTTTC |
| Transcription  activation in protoplast  (pRTBD) | ClNAC68^1-319^  ClNAC68^1-197^  ClNAC68^198-319^  ClActin | F:ctctctagaactagtggatccATGACTTGGTGCAGTGGCTCG  R:gcttttttggatatcgaattcTCAGATTTTCCTGTCGAGCTTTC  F:ctctctagaactagtggatccATGACTTGGTGCAGTGGCTCG  R:gcttttttggatatcgaattcGAGGTGGTATTGGTGCATGACC  F:ctctctagaactagtggatccGGCAACAACGAGGAAGAGAAAG  R:gcttttttggatatcgaattcTCAGATTTTCCTGTCGAGCTTTC  F:ctctctagaactagtggatccATGGCTGATGCTGAGGATATCCA  R:gcttttttggatatcgaattcTTAGAAGCACTTCCTGTGGACAA |
| Knockout of ClNAC68  (pBSE401) | ClNAC68 | BsF:ATATATGGTCTCGATTGCCGTCCGATGCCCATCCTGGTT  F0:TGCCGTCCGATGCCCATCCTGGTTTTAGAGCTAGAAATAGC  R0:AACGGCAAATCATGAATCCCAGCAATCTCTTAGTCGACTCTAC  BsR:ATTATTGGTCTCGAAACGGCAAATCATGAATCCCAGC |
| Primers for detecting mutant plants | ClNAC68 | F: TTCTTATGTTGGAGTGGGAATTAGC  R: AAAGAAGGGTACAGACCTGGAAGTT |
| qPCR of the target gene of ClNAC68 | ClINV  Cla97C10G201810  ClGH3.6  Cla97C05G096220  *ClACTIN*  Cla97C02G026960 | F: CATCAACTCCAGCTCCTTCC  R:ATTCGTGTCAACCCCACTTC  F: AGAGTCTCTCAACGCCGTGT  R: AACCTGGAACCCATTTAGGG  F: CCTACAACTCAATTATGAAGTGTG  R: GAAATCCACATCTGCTGGAAGGTG |
| EMSA assay  NAC binding sites were signed red | ClINV P1  ClINV P2  ClGH3.6 | F:AGTTTGGACTCCTTTCGTGGCTTGGTTAGTCTTAGT  R:ACTAAGACTAACCAAGCCACGAAAGGAGTCCAAACT  F:CTCTTCTCCACACGCTATACCCTGAGTGGTTTA  R:TAAACCACTCAGGGTATAGCGTGTGGAGAAGAG  F:TATTCTTCTTCCGTGTAGAAGATGACGAGAGTG  R:CACTCTCGTCATCTTCTACACGGAAGAAGAATA |
| Transient assay for luciferase | ClNAC68  ProClINV  ProClGH3.6 | F:cgctctagaactagtggatccATGACTTGGTGCAGTGGCTCG  R:gataagcttgatatcgaattcGATTTTCCTGTCGAGCTTTC  F:gtcgacggtatcgataagcttTCTAAAGGGAATCTAAATAGCTAGTTTTG  R:cgctctagaactagtggatccTTATGGTTACCAAGTCAAATTTGTATTT  F:gtcgacggtatcgataagcCCATAACATAGAATCCTGACACAAAAT  R:cgctctagaactagtggatccTTGTATCCTTTAAATTAAATATCACCACA |


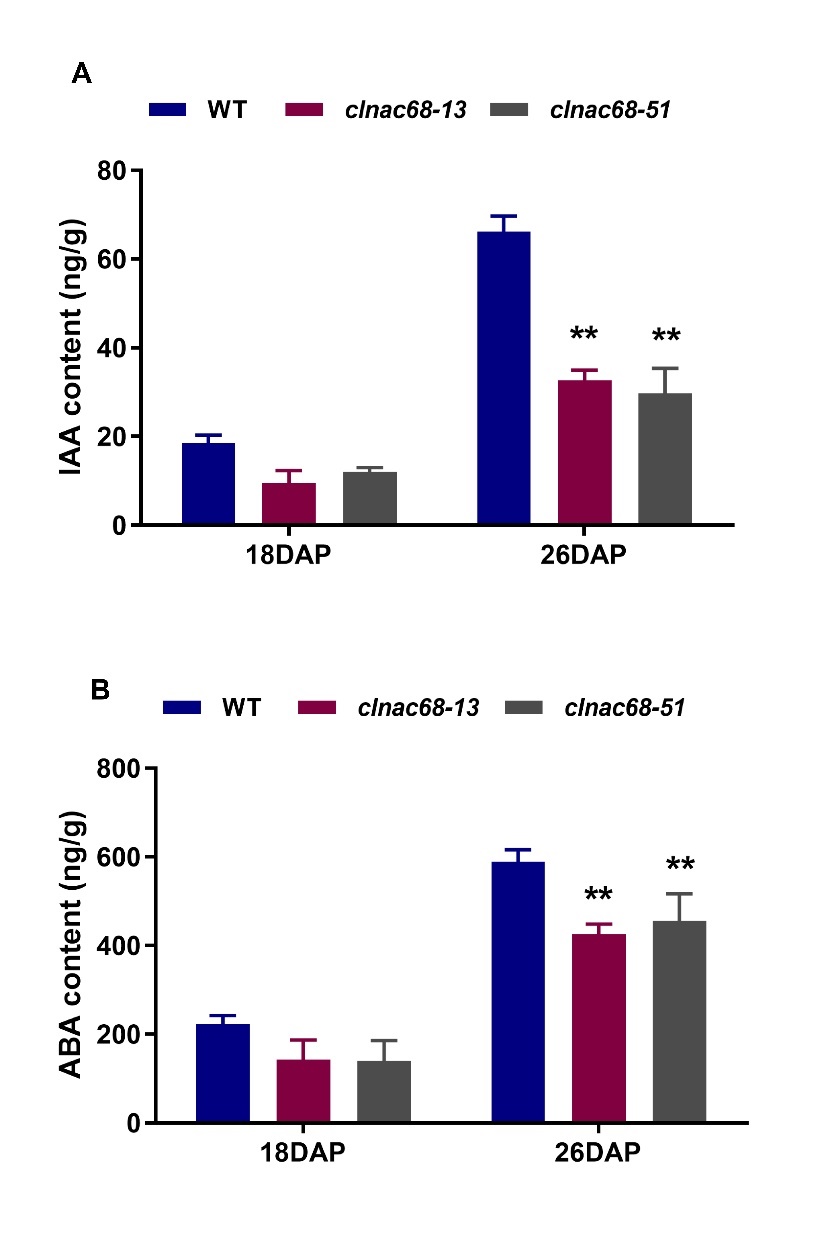


Figure S1. IAA and ABA contents in WT and ClNAC68 mutant fruits at 18 and 26 DAP. A. IAA content. B. ABA content. Asterisks denote Two-way ANOVA significance compared with WT plants at different developmental stage: **p*<0.05, ***p*<0.01, ****p*<0.001.


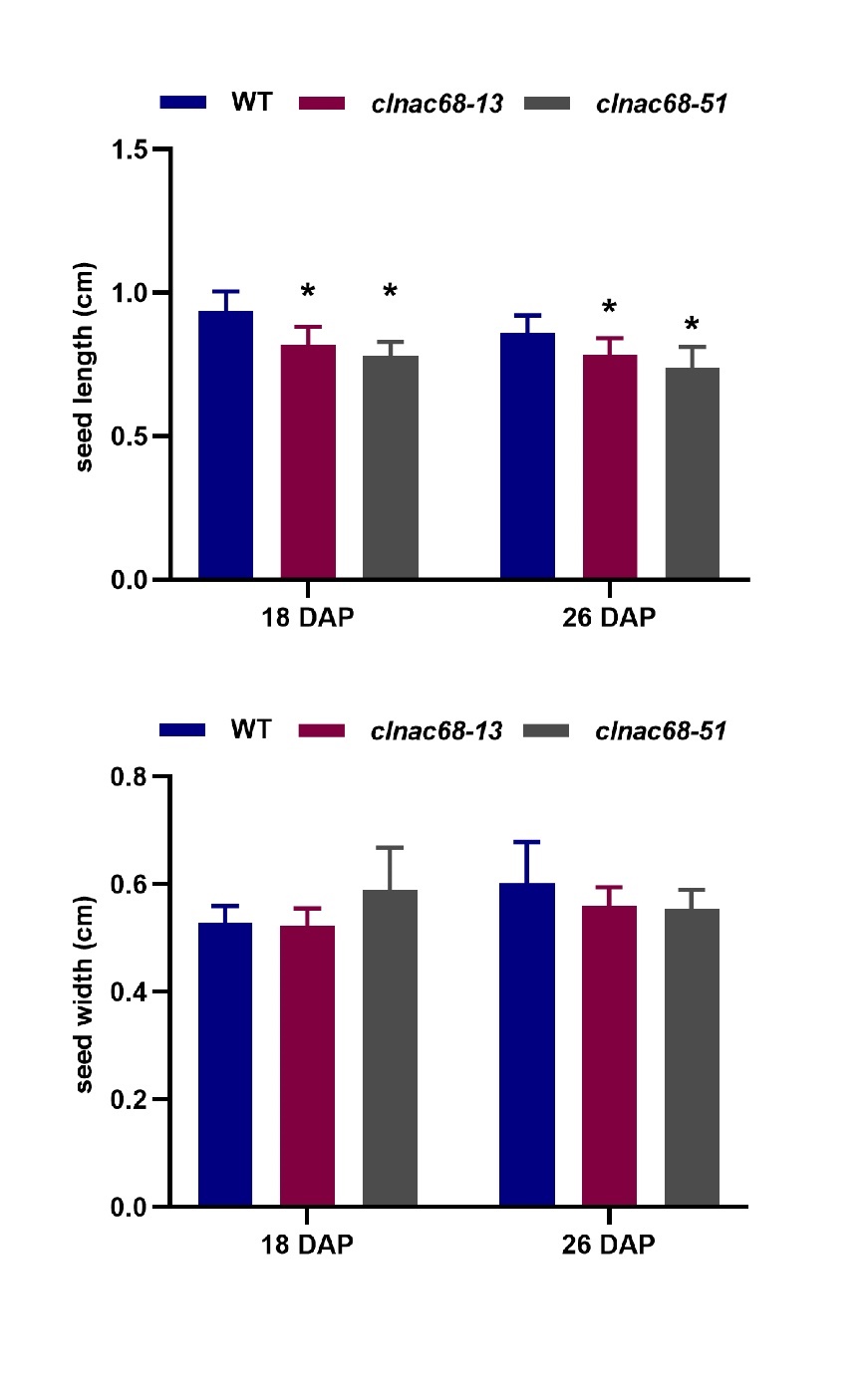


Figure S2. Seed length and seed width in WT and ClNAC68 mutant fruits at 18 and 26 DAP. A. Seed length. B. Seed width. Asterisks denote Two-way ANOVA significance compared with WT plants at different developmental stage: **p*<0.05, ***p*<0.01, ****p*<0.001.


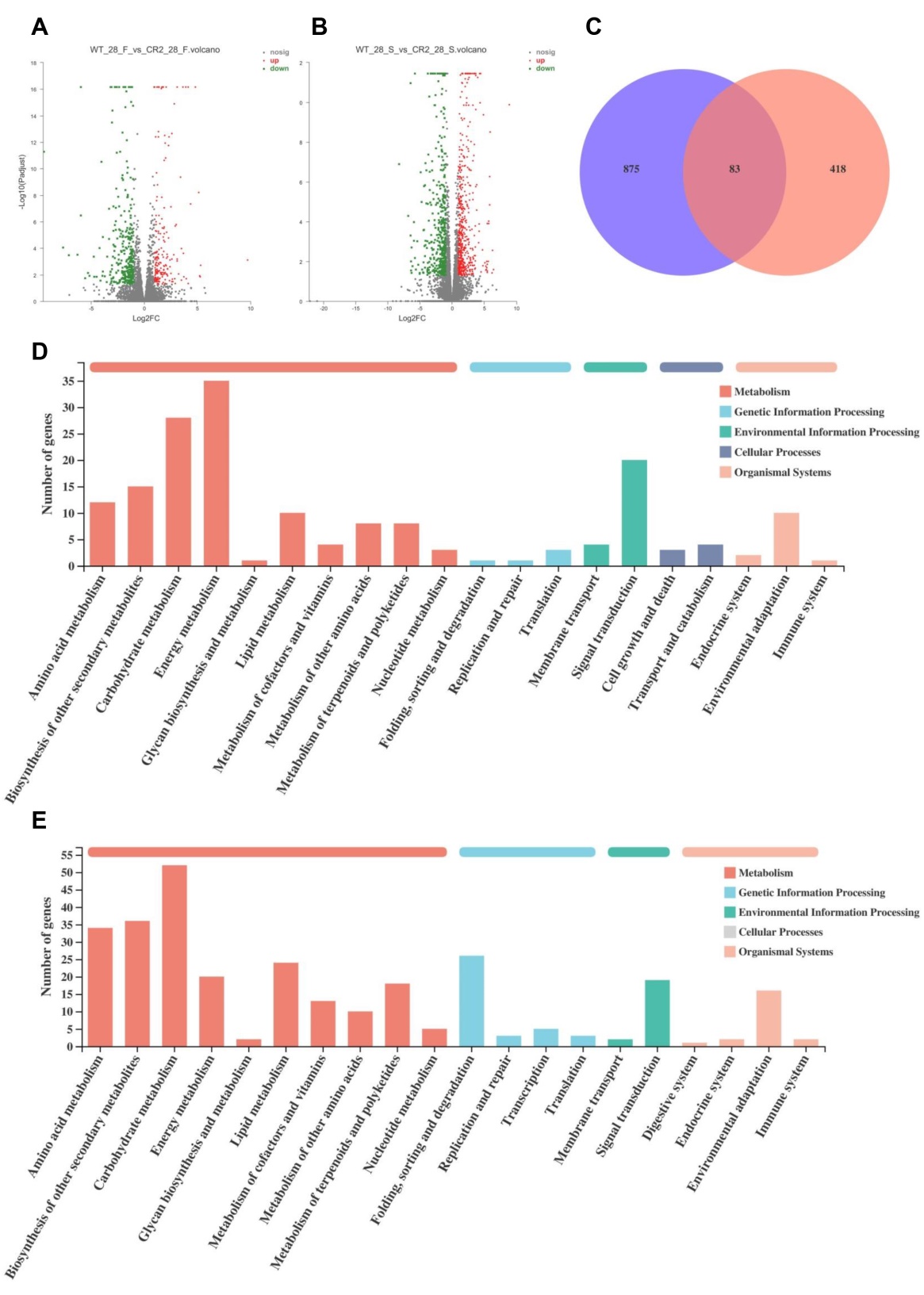


Figure S3. Volcano and KEGG analysis of DEGs in WT and ClNAC68 mutant lines at 26 DAP. A-B. Volcano analysis of DEGs in flesh (A) and seed (B) at 26 DAP. C. Venn analysis of DEGs in flesh and seed. D-E. KEGG analysis of DEGs in flesh (D) and seed (E) at 26 DAP.


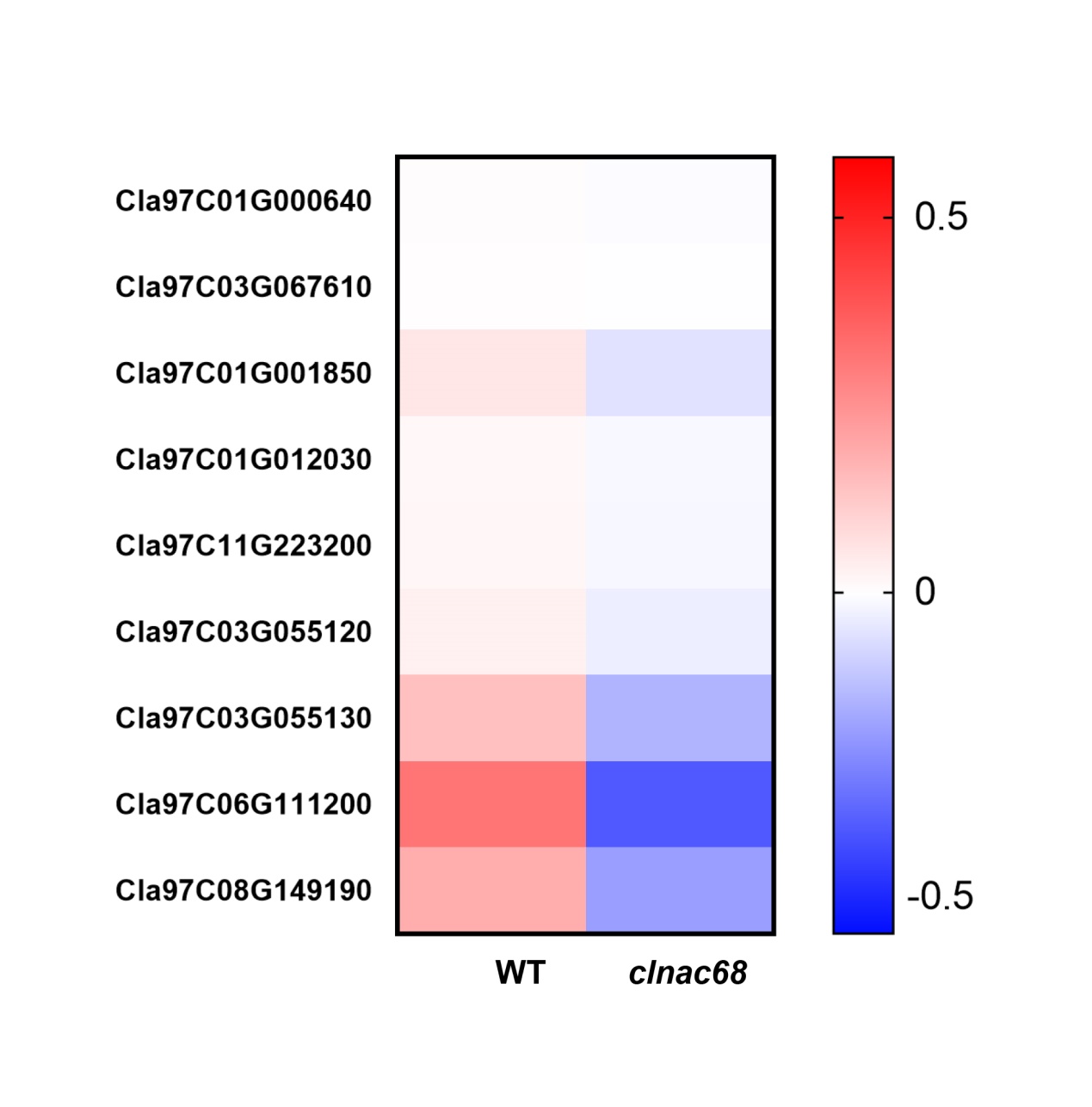


Figure S4. Heatmap analysis of *SWEET* genes in WT and *ClNAC68* mutant fruits at 26 DAP.


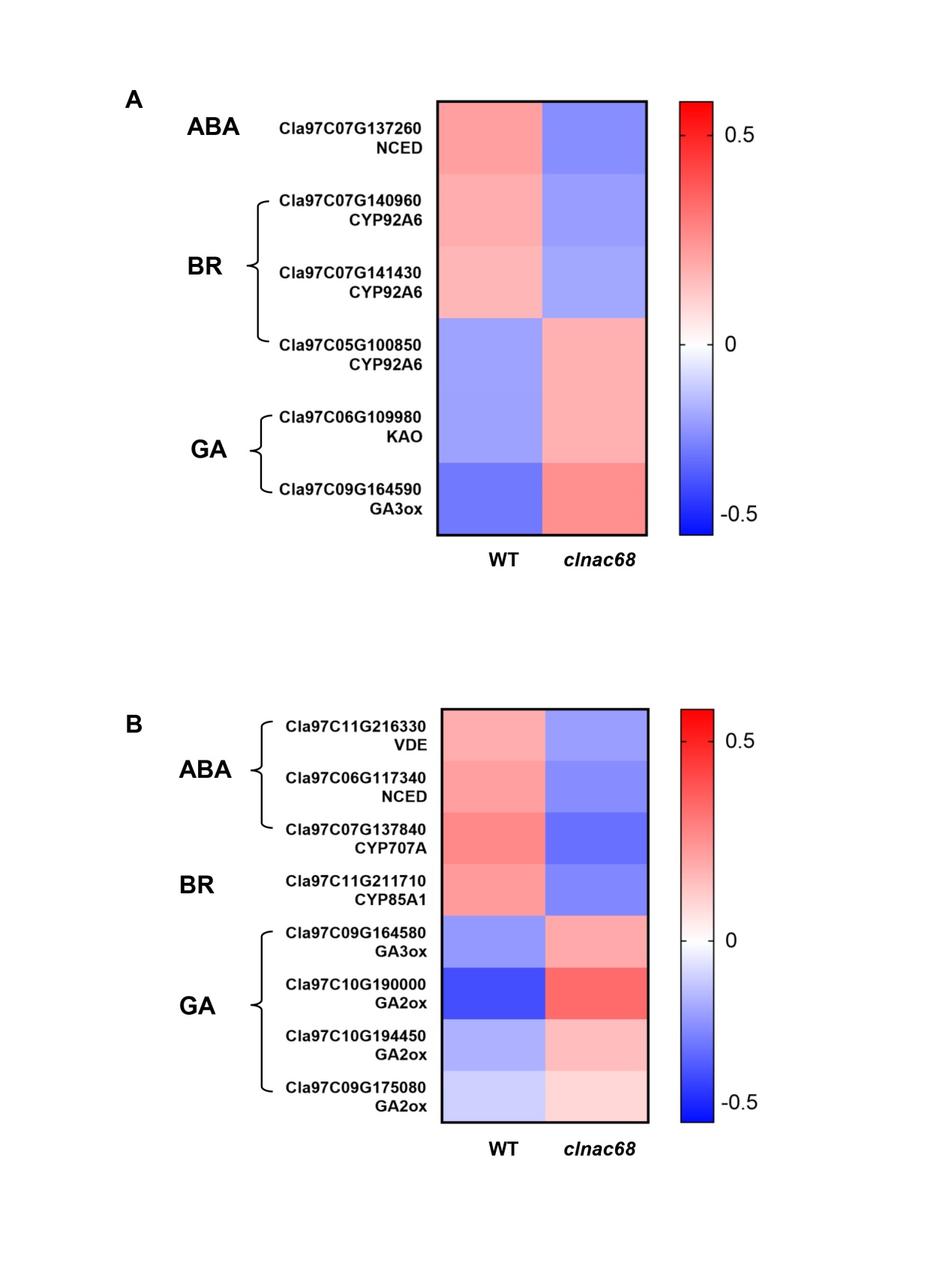
Figure S5. Heatmap analysis of the DEGs in hormone biosynthesis pathway in WT and *ClNAC68* mutant lines at 26 DAP. A. DEGs in flesh. B. DEGs in seed.
